# Prevalence estimates of HIV, syphilis, hepatitis B and C among female sex workers (FSW) in Brazil, 2016

**DOI:** 10.1097/MD.0000000000009218

**Published:** 2018-05-25

**Authors:** Orlando da Costa Ferreira-Júnior, Mark Drew Crosland Guimarães, Giseli Nogueira Damacena, Wanessa da Silva de Almeida, Paulo Roberto Borges de Souza-Júnior, Célia Landmann Szwarcwald

**Affiliations:** a Institute of Biology, Federal University of Rio de Janeiro, Rio de Janeiro; b Federal University of Minas Gerais, Belo Horizonte, Minas Gerais; c Health Information Laboratory, Institute of Communication and Scientific and Technological Information in Health, Oswaldo Cruz Foundation, Rio de Janeiro, Brazil.

**Keywords:** Brazil, co-infections, female sex workers, HBV, HCV, HIV, prevalence, respondent-driven sampling, syphilis

## Abstract

**Background:**

Female sex workers (FSW) bear a high burden of sexually transmitted infections (STI). In this paper, we estimate the prevalence of human immunodeficiency virus (HIV), HBV = hepatitis B virus (HBV), HCV = hepatitis C virus (HCV), syphilis and co-infections in the second Biological and Behavioral Surveillance Survey among FSW in Brazil.

**Method:**

The survey was conducted in 12 Brazilian cities from July to November 2016. We used respondent-driven sampling (RDS) to recruit 350 FSW in each city. Rapid tests were used for screening HIV, syphilis, HCV, and HBV. Confirmatory tests were performed on all samples with reactive rapid test result. All testing algorithms and interpretations were done according to the recommendations of the Department of STI/AIDS and viral hepatitis, Ministry of Health. The STI diagnoses were given by: confirmed HIV infection by a positive result on Western blot; active syphilis infection, defined by a RPR titer equal or greater than 1/8; viremia period of HBV and HCV infections, characterized by a detectable (or quantifiable) viral load. Prevalence estimates and standard errors were calculated using statistical procedures suitable for data collected by RDS.

**Results:**

Excluding the seeds, 4245 FSW were enrolled. Prevalence estimates were: HIV 5.3% (95% CI: 4.4%–6.2%); active syphilis 8.5% (95% CI: 7.3%–9.7%); HBV 0.4% (95% CI: 0.2%–0.7%); and, HCV 0.9% (95% CI: 0.6%–1.3%). Among the 4154 FSW tested for the 4 infections, 13.3%; (95% CI 12.0%–14.8%) were diagnosed with at least one of the infections, of which 87.6% (95% CI: 83.3%–90.9%) had single infections. The prevalence of HIV/syphilis co-infection was 1.09% (95% CI: 0.7%– 1.6%) and of HIV/HCV or HBV infections was 0.4% (95% CI: 0.2%–0.7%).

**Conclusions:**

Our results reveal the need to conduct more studies to estimate the prevalence of STI and co-infections among FSW in Brazil. Longitudinal trends in the prevalence estimates of HIV and other STI provide information to monitor changes in this high-risk population. Additionally, the study highlights the importance of measuring the hepatitis burden among FSW living with HIV, and the need of including FSW in all aspects of STI prevention, care, and treatment programs.

## 1 Introduction

The AIDS epidemic in Brazil remains concentrated, with a stable HIV prevalence in the general population around 0.6%,^[1]^ although geographical variation has been demonstrated and higher values are found in the South and Southeast regions of the country.^[2]^ Key populations with high HIV prevalence are men who have sex with men, injecting drug users and female sex workers (FSW).

Monitoring sexual practices of key population is important as changes in the patterns of sexual relationships among members of these groups may change the pattern of spread of HIV/AIDS in the general population.^[3]^ In particular, FSW continue to bear a high burden of HIV infection in many countries and are an important target population for a public health response to HIV/AIDS. However, this high risk group is often small in number and difficult to be accessed, in part because behaviors associated with FSW are highly stigmatized.^[4]^

Over time, various strategies have been suggested to slow down the AIDS epidemic in the world. In 2001, as a result of the global consensus aimed at slowing the HIV/AIDS epidemic, Brazil and 188 other countries signed the Declaration of Commitment during the 26th Special Session of the United Nations General Assembly (UNGASS), when a set of indicators was adopted to monitor the epidemic progress. Subsequently, in 2005, this set of indicators was reformulated, emphasizing the importance of monitoring groups at higher risk of HIV in countries with concentrated epidemics.^[5]^ Nowadays, the reduction in the number of new HIV infections in key populations is part of the sustainable development goals.^[6]^

Difficulties in monitoring sexual risk and HIV prevalence among higher risk groups have led to the development of specific sampling methods to collect information on hard-to-reach populations. One of these methods is respondent-driven sampling (RDS)^[7]^ a probability-based, chain recruitment sampling method that has been used in several countries among hard-to-reach populations, including FSW.^[8–13]^

In Brazil, efforts have been made to conduct a series of studies to characterize practices and risk behaviors among most-at-risk populations to HIV infection at the national level. The Brazilian Department of STI/AIDS and Viral Hepatitis, Ministry of Health (DIAHV/MoH) has adopted the RDS methodology^[7]^ to provide estimates of the prevalence and associated risk behaviors for HIV and syphilis among men who have sex with men,^[14]^ people who inject drugs,^[15]^ and FSW since 2009.^[16]^

This article builds on previous RDS study conducted in 10 Brazilian cities, in 2009, when baseline prevalence estimates of HIV and syphilis among FSW were estimated.^[16]^ The present study, conducted in 2016, aims to estimate the baseline prevalence of hepatitis B virus (HBV) and hepatitis C virus (HCV) and continue to evaluate HIV and syphilis prevalence estimates after 7 years from the first study.

## 2 Methods

### 2.1 Study design and eligibility

This Biological and Behavioral Surveillance Survey (BBSS) was conducted in 12 Brazilian cities from July to November 2016. The cities (aggregated by regions) are: Porto Alegre and Curitiba in the South; São Paulo, Rio de Janeiro and Belo Horizonte in the Southeast; Brasília and Campo Grande in the Central-West; Salvador, Recife, and Fortaleza in the Northeast; and Belém and Manaus in the North. The target number of FSW per city was 350. The decisions regarding which cities and sample size for each city to include in BBSS were made by the Department of STI, HIV/AIDS, and Viral Hepatitis, Ministry of Health (DIAHV/MoH) according to both, geographical criteria (5 Brazilian regions) and the epidemiologic relevance of the HIV/AIDS epidemic in Brazil. Eligibility criteria to participate in the study were the following: being at least 18 years of age, working as a sex worker in one of the municipalities of the study, having traded sex for money in the past 4 months, to assure recent sex work activity in the city; and present a valid coupon to participate. With a slight modification, this study follows the same BBSS protocol used to estimate the prevalence of HIV and syphilis in Brazilians FSW, that took place in 2009.^[16]^

### 2.2 Sampling methods

RDS data were collected through a peer recruitment mechanism in which current participants recruit future participants. For each site, 5 to 10 initial participants—referred to as seeds—were purposively chosen on the basis of a strong social network with the FSW population of the city. Each seed received 3 invitations (coupons) to give to other sex workers known to them. The recruits of the seeds in the survey were the first wave of the study. After participating in the interview, they received 3 coupons to distribute. This process was repeated until the sample size was achieved in each location. The chains were traceable with the coupon serial numbers.

Participants also answered to a questionnaire divided by modules to collect information on sociodemographic data, knowledge of HIV and other STI transmission, sexual behavior, previous HIV testing, history of STI, alcohol and illicit drug use, access to health care and prevention activities, discrimination, and violence.

The research project was approved by the Ethics Committee of the Oswaldo Cruz Foundation (Protocol 1.338.989) and participants signed an informed consent. All parts of the survey were confidential and only the coupon number of each participant linked the questionnaires with the blood samples. All participants could refuse to continue participating in the study whenever they wished.

### 2.3 Laboratory assays

HIV, HBV, HCV, and syphilis tests were conducted using rapid tests, according to the recommendations of the DIAHV/MoH.^[17]^ After signing the informed consent all participants received pretest orientation about the tests to be performed and post-test counseling after receiving the results. Those who had a reactive test result were referred to public health services for follow-up.

At the study sites, a phlebotomist collected 2 ethylenediaminetetraacetic acid (EDTA) tubes, each containing 5 mL of venous blood. One EDTA tube was used for HIV, HBV, HCV, and syphilis rapid testing, while participants were answering the study questionnaire. The second EDTA tube, containing a gel plug, was centrifuged to separate plasma and was kept frozen at −20^o^C until transfer to the Molecular Virology Laboratory of the Federal University of Rio de Janeiro, Rio de Janeiro, where all confirmatory tests for HIV, HBV, HCV, and syphilis were performed. No confirmatory assay was pursued when rapid tests indicated nonreactive results. If a participant refused to collect venous blood, the research team offered the option to perform rapid tests by finger pricking. In this case, no confirmatory assays were performed in the survey even if any of the rapid tests scored reactive results. However, participants who tested positive for any of the rapid tests were referred to public health systems for follow-up.

Whole blood specimens were screened for HIV, HBV, HCV, and syphilis antibodies with the following assays: HIV (HIV Test Bioeasy, Standard Diagnostic Inc, Korea and ABON HIV 1/2/O Tri-Line Human Immunodeficiency Virus Rapid Test Device, China), HBV (Vikia HBsAg, BioMérieux SA, France), HCV (ALERE HCV, Standard Diagnostic Inc., Korea) and syphilis, treponemal assay (SD BIOLINE Syphilis 3.0, Standard Diagnostic Inc., Korea). In accordance to the DIAHV recommendation, a reactive result on the initial HIV rapid test (HIV Test Bioeasy, Standard Diagnostic Inc., Korea) should be followed by a second HIV rapid test, from a different manufacturer (ABON HIV 1/2/O Tri-Line Human Immunodeficiency Virus Rapid Test Device, China). Samples reactive on any of the 2 rapid tests were further submitted to confirmatory assays.

Confirmatory tests using plasma samples were: HIV-1 Western Blot for HIV (Cambridge Biotech HIV-1 Western Blot Kit, Maxim Biomedical, Inc.); viral load for both HBV (Abbott RealTime HBV viral load assay, Abbott Laboratories) and HCV (Abbott RealTime HCV viral load assay, Abbott Laboratories) and; rapid plasma reagin (RPR) for syphilis (RPR Sífilis, WAMA *Diagnóstica*, Brazil).

The diagnostic algorithms and interpretations used in this study were in accordance to the DIAHV/MoH recommendations, as follows: confirmed HIV infection by a positive result on Western blot; active syphilis infection, defined by a RPR titer equal or greater than 1/8; and viremia period of HBV and HCV infections, characterized by a detectable (or quantifiable) viral load.

### 2.4 Data analysis

The estimators of the prevalence and standard errors were calculated using statistical procedures suitable for analysis of data collected by RDS. We considered the effect of homophily, the intracluster correlation of participants recruited by the same person as well as the unequal selection probabilities. The prevalence estimates and their corresponding standard errors were based on the estimation of transition probabilities from one state (positive) to another (negative) and vice-versa.^[16]^

### 2.5 Ethical considerations

The research project was approved by the Ethics Committee of the Oswaldo Cruz Foundation (Protocol 1.338.989) and followed the National Health Council guidelines, assuring the subjects’ voluntariness, anonymity and possibility of withdrawal at any moment in the study, through the signing of a Consent Form.

## 3 Results

Overall, the survey successfully interviewed 4328 FSW during a 5-month period. Excluding the 83 seeds, the number of participants by city was as follows: Belo Horizonte (343), Belem (345), Brasilia (354), Curitiba (341), Campo Grande (346), Fortaleza (346), Manaus (353), Porto Alegre (347), Recife (349), Rio de Janeiro (422), São Paulo (359), and Salvador (340) totalizing 4245 FSW.

Among the 4245 recruited FSW, 4190 (98.7%) accepted to collect venous blood for the rapid test screenings followed by confirmatory testing among those positive in any of the tests. Around 47 (1.2%) only agreed with finger pricking for screening procedures, precluding the execution of confirmatory tests. Only 8 participants refused to collect any source of blood sample for testing. For standardization purposes, in this analysis, we only considered the participants who accepted to collect venous blood.

Table 1 shows the prevalence estimates for the 4 infectious disease agents among FSW in the 2016 study. For comparison, the estimates for HIV and syphilis prevalence obtained in the 2009 study are also presented in Table 1.^[16]^ In the 2009, BBSS, 2505 FSW were tested only for HIV and syphilis.

**Table 1:**
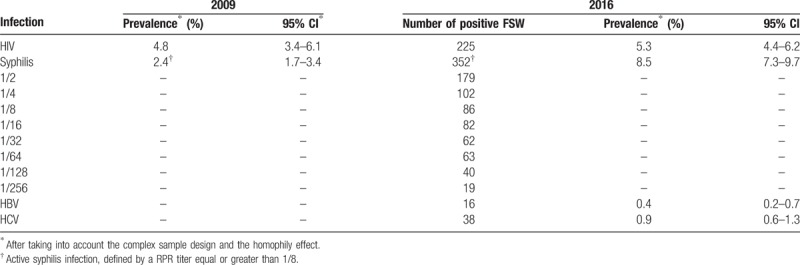
HIV, syphilis, HBV, and HCV prevalence estimates among FSW, Brazil, 2009 and 2016.

The estimate of HIV prevalence among FSW in the current study was 5.3% (95% CI: 4.4%–6.2%) a value not statistically different from the 2009 estimate of 4.8%—(95% CI: 3.4%–6.1%). However, among FSW, the estimated prevalence of active syphilis in 2016 (8.5%; 95% CI: 7.3%–9.7%) was 3.5 times higher than that of 2009 (2.4%; 95% CI: 1.7%–3.4%). In addition, in the current study the estimates of HBV and HCV prevalence were 0.4% (95% CI: 0.2%–0.7%) and 0.9% (95% CI: 0.6%–1.3%), respectively, and they represent the baseline prevalence rates for FSW in Brazil for future comparisons.

Table 2 shows different combinations of co-infections among the surveyed FSW. Among the 4154 FSW tested for the 4 infections, 13.3%; (95% CI: 12.0%–14.8%) were diagnosed with at least 1 of the infectious agents, of which 87.5% had single infections, 11.4% had dual infections, and only 1.1% had triple infections. No FSW was infected by the 4 STI simultaneously. It is noteworthy that the prevalence of HIV and syphilis co-infection (1.1%) was greater than the sum of the 2 individual estimates for HBV and HCV. The estimated prevalence of HIV with HCV or HBV infections was 0.4% (95% CI: 0.2%–0.7%). We did not observe any concomitant HBV and HCV viremia among the participants.

**Table 2:**
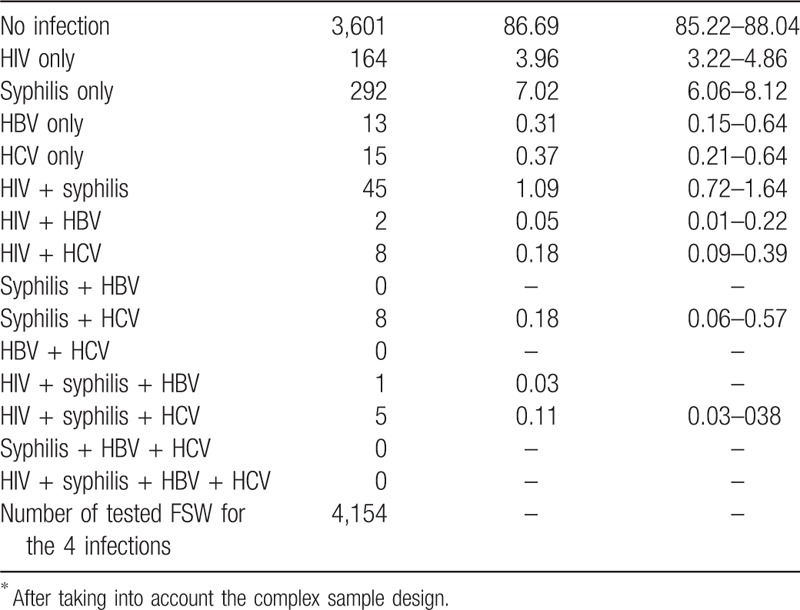
Prevalence of the 4 STI co-infections among FSW, Brazil, 2016.

## 4 Discussion

In this second biological and behavioral survey among FSW in Brazil, the prevalence estimates expanded from HIV and syphilis to include HBV and HCV. This article focused on estimating prevalence rates for these 4 sexually transmitted infections and co-infections in this most-at-risk group. The correlates of HIV infection and of the other STI will be subject of other publications.

We indicate that the HIV prevalence among FSW in Brazil has remained stable during the 7-year period between the 2 RDS studies conducted in Brazil in 2009 and 2016.^[16]^ However, this prevalence is still 1 order of magnitude higher than the 0.4% estimate found in pregnant women aged 15 to 49 years old.^[18]^ These results corroborate the findings of a meta-analysis study showing that FSW in low and middle income countries are 13.5 times more likely to be HIV infected than other women.^[19]^

Overall, the results of the current study reinforce that interventions targeting FSW should continue to be supported, including HIV testing, condom promotion, syndromic management of STIs, and education on how to control sex work-related HIV transmission besides access and adherence to antiretroviral therapy.^[20–22]^ In this regard, HIV testing becomes particularly relevant as a point of entry to health care and treatment. There is an extensive network of services that could provide HIV testing and counseling to FSW in Brazil. Testing sites vary from conventional health centers to mobile testing units in streets—where services are provided during times and locations convenient to key populations. Expanding HIV testing to this population offers the benefit of diagnosing large percentage of HIV-infected FSW unaware of their HIV infection and the possibility of early treatment.^[23,24]^

Regarding syphilis, the estimated prevalence of 8.5% represents an increase in syphilis burden among FSW—it is more than 3.5 times than the estimated prevalence of 2.4% found in the 2009 RDS study.^[16]^ These data parallel the situation found among pregnant women in Brazil with a considerable growth in the detection rate of syphilis.^[25]^

Given the high prevalence of syphilis infection in this population and the concern of potential syphilis transmission among FSW and their partners, a national control plan for syphilis must be on sight. Continued surveillance among FSW to monitor trends of prevalence and behavioral risk factors for syphilis, in addition to availability of rapid point-of-care tests in public clinics to promote timely diagnosis are important aspects to be addressed.^[26]^ Equally relevant, health staff should be devoid of stigma, as it hampers access to treatment and health care efforts for FSW, and inhibits periodic visits to health units where regular testing and clinical examinations could anticipate diagnosis and treatment of STI in this most-at-risk population group.^[27]^

Moreover, the increased trend in the syphilis positivity rate suggests a shift in STI risks among FSW over time. A combination approach to the current implementation process of pre-exposure prophylaxis (PrEP) program for HIV in Brazil (i.e. antiretroviral prescription and HIV testing) is highly recommended, including reinforcement of use and distribution of condom as well as regular testing for syphilis and syndromic assessment of other STI.^[28,29]^

Information on the prevalence of multiple co-infections with different STIs among FSW is still limited.^[30,31]^ Monitoring viral hepatitis and co-infection with HIV among key populations is a relatively recent public health concern, mostly due to the recognition that many people living with HIV receiving antiretroviral therapy are dying from liver disease resulting from untreated viral hepatitis.^[32]^ From the public health perspective, new effective drugs against all HCV genotypes and the possibility to adjust HIV drug regimens to include treatment for HBV have changed this scenario.^[33]^

In this study, nearly 13% FSW were diagnosed with at least one STI, and among those, 1.1% with HIV and syphilis co-infection, and 0.4% with HIV and viral hepatitis (HCV or HBV) co-infection. Sexual transmission of HBV infection has been known for a long time.^[34–36]^ Drug use, mostly by needle sharing, is the main risk factor of contracting HCV as well as HBV.^[37]^ However, general drug use may disinhibit risk perceptions and promote risky behaviors.^[38]^ The vicious drug-sex circle frequently culminates in exchanging sex for drugs.^[39]^

Measuring the hepatitis burden among key populations living with HIV is important to improve viral hepatitis program, including the introduction of appropriate treatment. Furthermore, the implementation of rapid testing sites for both, HBV and HCV, is essential as HIV-positive individuals co-infected with HBV or HCV suffer from liver pathology associated with morbidity and mortality.^[40]^ Infected FSW will benefit from treatment of either viruses and, in addition, HBV negative women can be referred and enrolled in HBV immunization program.^[41]^

In view of the lack of data on STI co-infections among FSW in Brazil, our results reveal the need to conduct more studies to estimate the prevalence of HIV and co-infections with other STI in this high-risk population. As prevalence reflects both incidence and survival, documenting HIV prevalence over time can provide insight into the impact of combined prevention approaches, such as pre-exposure and post-exposure prophylaxis, test and treat strategy, and treatment of STI.^[42]^ Additionally, the study highlights the importance of measuring the hepatitis burden among FSW living with HIV, and the need of including FSW in all aspects of STI prevention, care, and treatment programs.

## Author contributions

This manuscript has not been submitted or accepted for publication elsewhere. All authors contributed to the concept of the paper and data analysis. OCFJ, MDCG, and CLS were responsible for the writing of the final version of the manuscript and CLS, PRBSJ, GND, and WSA were responsible for the statistical analysis. All authors have read and approved the paper, have met the criteria for authorship as established by the International Committee of Medical Journal Editors, believe that the paper represents honest work, and are able to verify the validity of the results reported.

## Acknowledgments

The authors would like to express their gratitude to the participants of the study and to the local teams that carried out the fieldwork in the 12 cities. We are also grateful for the support of STI/HIV/Aids and Viral Hepatitis Department of the Brazilian Minister of Health. Additionally, we appreciate the support of The Brazilian FSW Group: Celia Landmann Szwarcwald, Paulo Roberto Borges de Souza Júnior, Orlando C. Ferreira Jr., Giseli Nogueira Damacena, Neide Gravato da Silva, Rita Bacuri, Helena Brigido, Hermelinda Maia Macena, Ana Brito, Inês Dourado, Mark Drew Crosland Guimarães, Wanessa da Silva de Almeida, Alexandre Grangeiro, Carla Luppi, Karin Regina Luhm, Isete Maria Stella, Adriana Varela Espinola, Tânia Varela, and Francisca Sueli da Silva.
